# A Galantamine–Curcumin Hybrid Decreases the Cytotoxicity of Amyloid-Beta Peptide on SH-SY5Y Cells

**DOI:** 10.3390/ijms22147592

**Published:** 2021-07-15

**Authors:** Kirilka Mladenova, Georgi Stavrakov, Irena Philipova, Mariyana Atanasova, Svetla Petrova, Jordan Doumanov, Irini Doytchinova

**Affiliations:** 1 Faculty of Biology, Sofia University, 1164 Sofia, Bulgaria; k_mladenova@biofac.uni-sofia.bg (K.M.); spetrova@biofac.uni-sofia.bg (S.P.); doumanov@biofac.uni-sofia.bg (J.D.); 2 Faculty of Pharmacy, Medical University of Sofia, 1000 Sofia, Bulgaria; stavrakov@pharmfac.mu-sofia.bg (G.S.); matanasova@pharmfac.mu-sofia.bg (M.A.); 3 Institute of Organic Chemistry with Centre of Phytochemistry, Bulgarian Academy of Sciences, 1113 Sofia, Bulgaria; irena@orgchm.bas.bg

**Keywords:** amyloid beta peptide, galantamine, curcumin, galantamine–curcumin hybrid, cytotoxicity, cytoprotection, SH-SY5Y cell line

## Abstract

Misfolded amyloid beta (Aβ) peptides aggregate and form neurotoxic oligomers. Membrane and mitochondrial damages, calcium dysregulation, oxidative stress, and fibril deposits are among the possible mechanisms of Aβ cytotoxicity. Galantamine (GAL) prevents apoptosis induced by Aβ mainly through the ability to stimulate allosterically the α7 nAChRs and to regulate the calcium cytosolic concentration. Here, we examined the cytoprotective effects of two GAL derivatives, namely compounds **4b** and **8**, against Aβ cytotoxicity on the human neuroblastoma cell line SH-SY5Y. The protective effects were tested at simultaneous administration, pre-incubation and post-incubation, with Aβ. GAL and curcumin (CU) were used in the study as reference compounds. It was found that **4b** protects cells in a similar mode as GAL, while compound **8** and CU potentiate the toxic effects of Aβ. Allosteric stimulation of α7 nAChRs is suggested as a possible mechanism of the cytoprotectivity of **4b**. These and previous findings characterize **4b** as a prospective non-toxic multi-target agent against neurodegenerative disorders with inhibitory activity on acetylcholinesterase, antioxidant, and cytoprotective properties.

## 1. Introduction

In recent years, cases of neurodegenerative diseases have increased significantly worldwide and in the next decades it will become the second leading cause of death, displacing cancer [1]. Among them, Alzheimer’s disease (AD) is the most common cause of dementia, affecting 17% of the population over the age of 75 [2]. The main symptoms of the disease range from memory deficiency with names and events, communication problems and disorientation, to total inability to perform basic activities of daily living [3]. The disease begins slowly, many years before the clinical manifestations, making it difficult for early diagnosis and prompt treatment of mild and moderate forms.

One of the main hallmarks of AD is the insoluble misfolded amyloid fibrils. They are formed gradually by aggregation of single monomers into nuclei with a hydrophobic core. The nuclei elongate to protofibrils and fibrils with cross-β conformation by attaching novel monomers [4]. The fibrils are arranged into amyloid plaques that cause cerebral angiopathy and neuronal loss. Inhibiting early amyloid aggregation via small molecules is one of the strategies for developing of anti-amyloid drugs [5].

The main drugs currently used for symptomatic treatment of mild and moderate forms of AD are inhibitors of the enzyme acetylcholinesterase (AChE) [6]. The enzyme AChE catalyses the hydrolysis of acetylcholine (ACh) to choline and acetic acid in the cholinergic synapses. AChE inhibition leads to ACh accumulation and a temporary delay in cognitive decline.

Galantamine (GAL) is one of the most widely used AChE inhibitors for the treatment of AD. Over the years it has been widely studied and has proven to be a drug with a multi-targeted action [7,8]. In addition to moderate AChE inhibitory activity, GAL binds allosterically to α7 nAChR and prevents apoptosis induced by β-amyloid and thapsigargin—a specific α7 nAChR antagonist—through increasing the density of α7 nAChRs and expression of the antiapoptotic protein Bcl-2 in human neuroblastoma cell line SH-SY5Y [9]. GAL blocks the Aβ-enhanced glutamate toxicity by inducing phosphorylation of the kinase effector Akt also mediated by α7 nAChRs [10]. Allosteric activation of α7 nAChRs is associated with GAL’s ability to restore the cholinergic cells in anti-NGF transgenic mice and to reduce the perivascular APP deposits [11].

GAL directly inhibits the aggregation of Aβ peptides and dramatically reduces the apoptosis of SH-SY5Y cells caused by Aβ_1–40_ and Aβ_1–42_ [12]. GAL binds to Aβ dimer and causes a significant conformational change at the turn region (Asp23-Gly29), leading to disruption between β-strands and prevention of neurotoxic oligomer formation [13]. GAL significantly decreases Aβ secretion by inhibiting BACE1 expression (at low concentrations) or by decreasing the amount of APP (at higher concentrations) [14]. Takata et al. [15] found that GAL facilitates Aβ clearance by sensitizing microglial α7 nAChRs to choline and inducing Ca^2+^ influx into microglial cells, followed by actin reorganization and Aβ phagocytosis.

**Figure 1:**
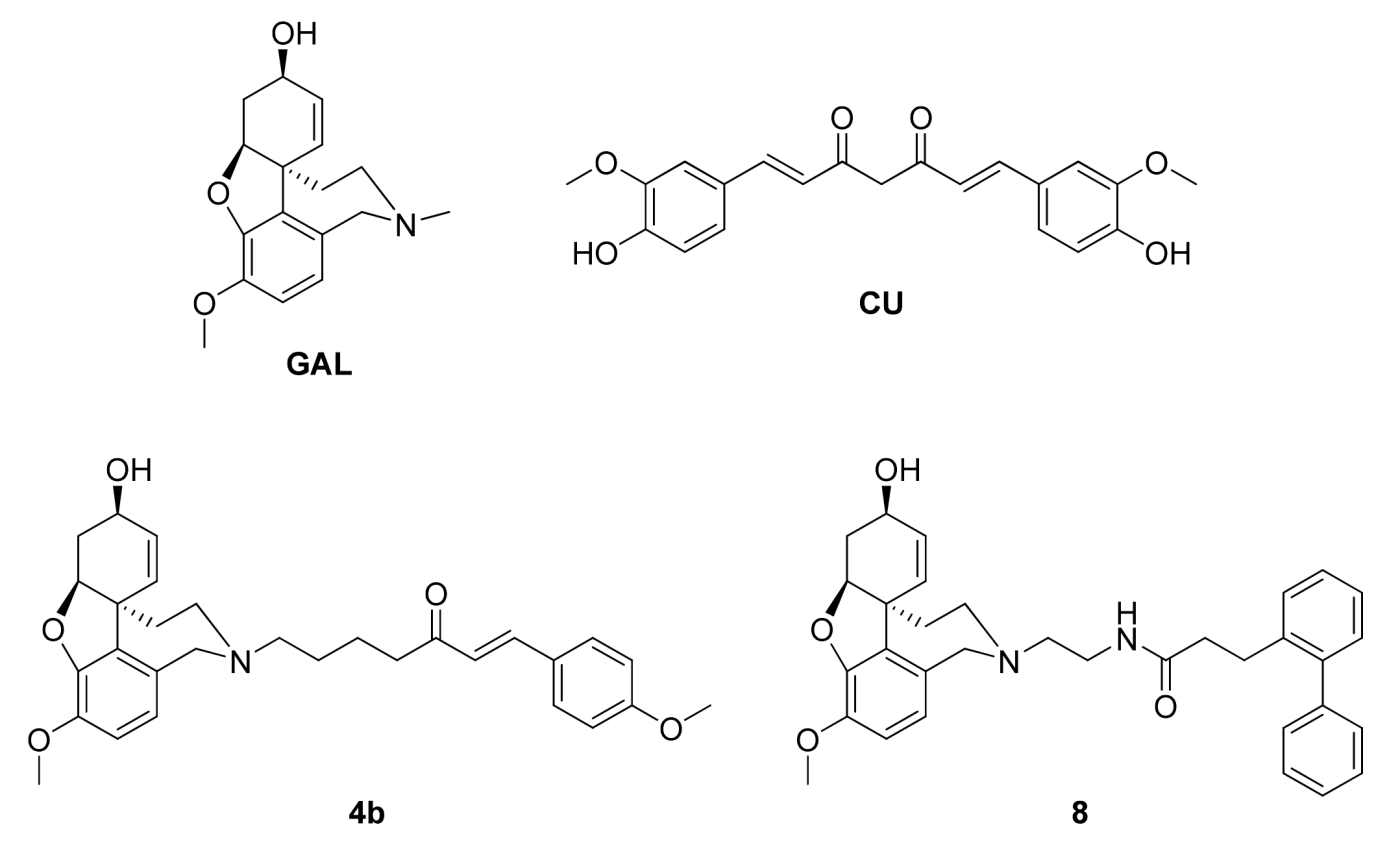
Structures of galantamine (GAL), curcumin (CU), their hybrid **4b,** and compound **8**. The IC_50_ values of the tested AChE inhibitors measured previously using Ellman’s method were: 0.020 µM for **4b** [16], 0.028 µM for **8** [17], 3.52 µM for GAL [16], and 67.69 µM for CU [16].

Recently, we designed two series of GAL derivatives. One of the series contains curcumin aromatic fragments [16], while the other one incorporates non-curcumin aromatic fragments [17]. Curcumin (CU) is a natural polyphenol with powerful antioxidant properties [18] and an ability to inhibit Aβ aggregation [19] and to reduce amyloid plaques [20]. The GAL derivatives were screened virtually via molecular docking and the most promising compounds were synthesized and tested for anti-AChE activity [16]. Five compounds from the GAL–CU series and one compound from GAL–non-CU series showed less toxicity than GAL and CU and higher activity than GAL. Here, we examined the most active compounds from both series, namely **4b** and **8** (Figure 1), for their ability to prevent the cytotoxic effects of Aβ peptides. GAL and CU were used in the study as controls. The IC_50_ values of the tested AChE inhibitors measured previously using Ellman’s method were: 0.020 µM for **4b** [16], 0.028 µM for **8** [17], 3.52 µM for GAL [16], and 67.69 µM for CU [16]. As GAL derivatives, compounds **4b** and **8** were expected also to reduce the toxic effects of Aβ. As the present study was a continuation of our previous research [16,21], we preferred to keep the original compound IDs.

## 2. Results

In order to examine the effects of the newly designed and synthesized AChE inhibitors on the toxicity caused by Aβ on SH-SY5Y cells, three experimental scenarios were tested. In the first scenario, the tested compounds were administered simultaneously with Aβ peptides. This test showed the immediate protection of the compounds against Aβ toxicity. In the second scenario, the cells were preincubated with the tested compounds for 24 h, followed by the addition of Aβ. The aim of this test was to examine the ability of the compounds to prevent Aβ cytotoxicity. In the third scenario, the cells were preincubated with Aβ peptides for 24 h and then the tested compounds were added. The results here were indicative for the ability of compounds to repair the damage caused by Aβ and to restore cell viability. In all tests, GAL and CU were used as reference compounds. Before applying the three scenarios, the effect on the cell viability of each studied compound was tested.

### 2.1. Cytotoxicity of Amyloid β Peptide

**Figure 2:**
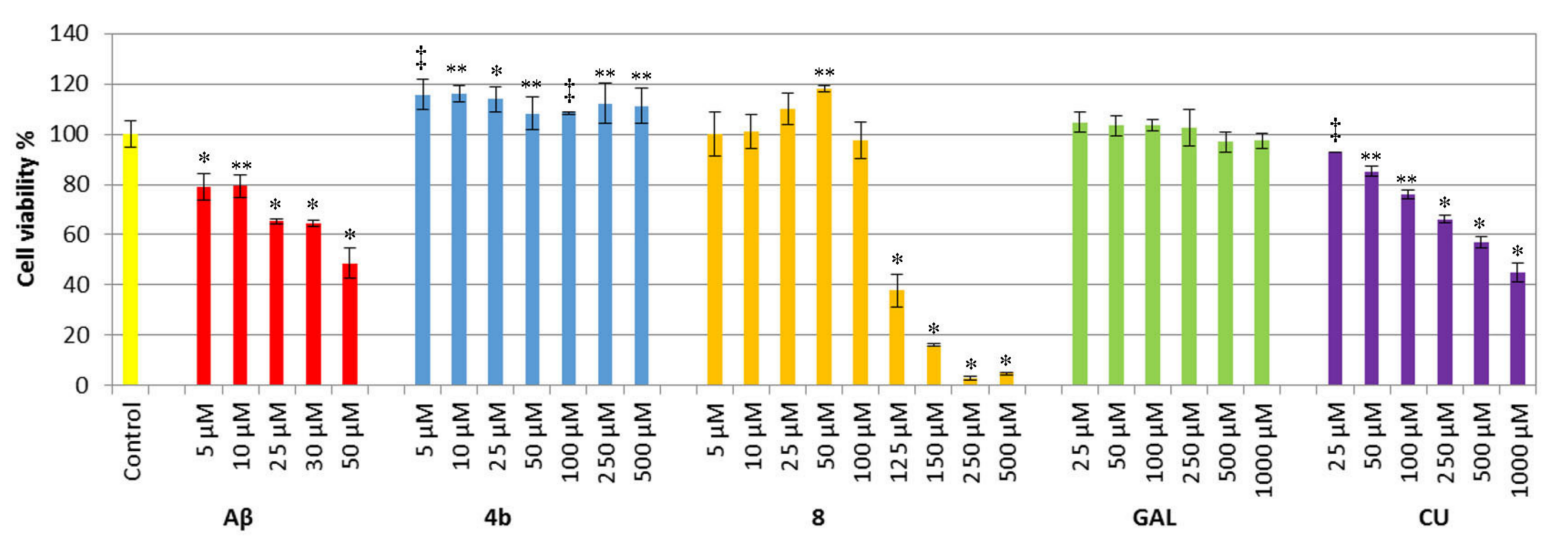
Cell viability (percentage) of SH-SY5Y cells treated for 48 h at 37 °C with different concentrations of Aβ, **4b**, **8**, GAL, and CU. Values are expressed as a mean ± SE, *n* = 3, *p*-values are presented as: * for *p* < 0.01, ** for *p* < 0.05, and ‡ for *p* ≤ 0.1.

The Aβ peptide administered in concentrations of 5–50 µM for 48 h at 37 °C showed dose-dependent cytotoxicity on SH-SY5Y cells (Figure 2). Even at the lowest concentration of 5 µM, Aβ decreased the cell viability by 21%. At concentrations of 25 µM and 30 µM, the cell viability decreased to 65%, and at 50 µM to 48%. Further, in the three experimental scenarios Aβ peptide was used at a concentration of 25 µM.

**Figure 3:**
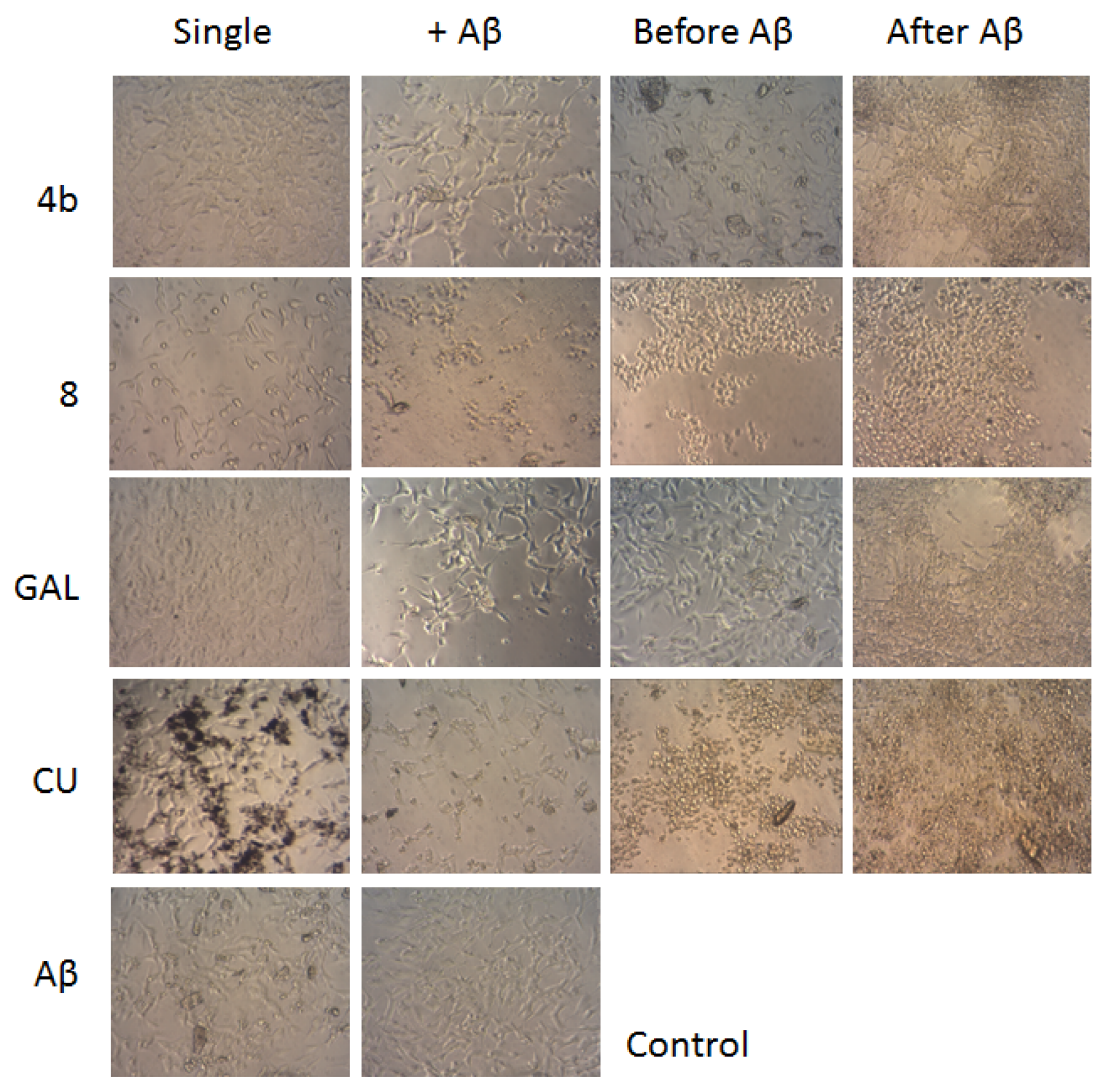
Images of SH-SY5Y cells taken 24 h after being exposed to single **4b**, **8**, GAL, and CU in 100 µM, Aβ in 25 µM, and by **4b**, **8**, GAL, and CU in 100 µM administered simultaneously with Aβ, before introduction of Aβ, and after introduction of Aβ.

The cytotoxic effect of Aβ on SH-SY5Y cells is visualized 24 h after being exposed to Aβ in Figure 3. The cells grown in the presence of Aβ showed separation from the neighbouring cells due to membrane retraction and fragmentation of cell monolayers, which caused a decrease in cell viability.

### 2.2. Cytotoxicity of the Tested AChE Inhibitors

The cytotoxicity values of the AChE inhibitors tested in the present study are given in Figure 2. Compound **4b** was non-toxic at concentrations of 5–500 µM. Even more, in the presence of **4b** the cell viability increased to 108–116% compared with control cells. Compound **8** was non-toxic at concentrations up to 50 µM. At higher concentrations, the toxicity increased dose-dependently, reaching 3% cell viability at 250 µM. GAL was non-toxic up to 250 µM. At 500 and 1000 µM, the cell viability decreased only by 3–4%. CU showed dose-dependent cytotoxicity in the range of 25–1000 µM. At 1000 µM, only 45% of the SH-SY5Y cells survived. Based on these observed single cytotoxicity levels, the inhibitors were further tested in the range of 100–500 µM in three concentrations: 100, 250, and 500 μM. Being more toxic, compound **8** was tested in a lower range—from 50 to 150 μM: 50, 100, and 150 μM.

The morphology of SH-SY5Y cells in the presence of 100 µM of each inhibitor is shown in Figure 3. In the presence of **4b** and GAL, the cells proliferated, while in the presence of **8** and CU the number of cells decreased and they lost confluence.

### 2.3. Cytotoxicity of Amyloid β Peptides Given with the Tested AChE Inhibitors

**Figure 4:**
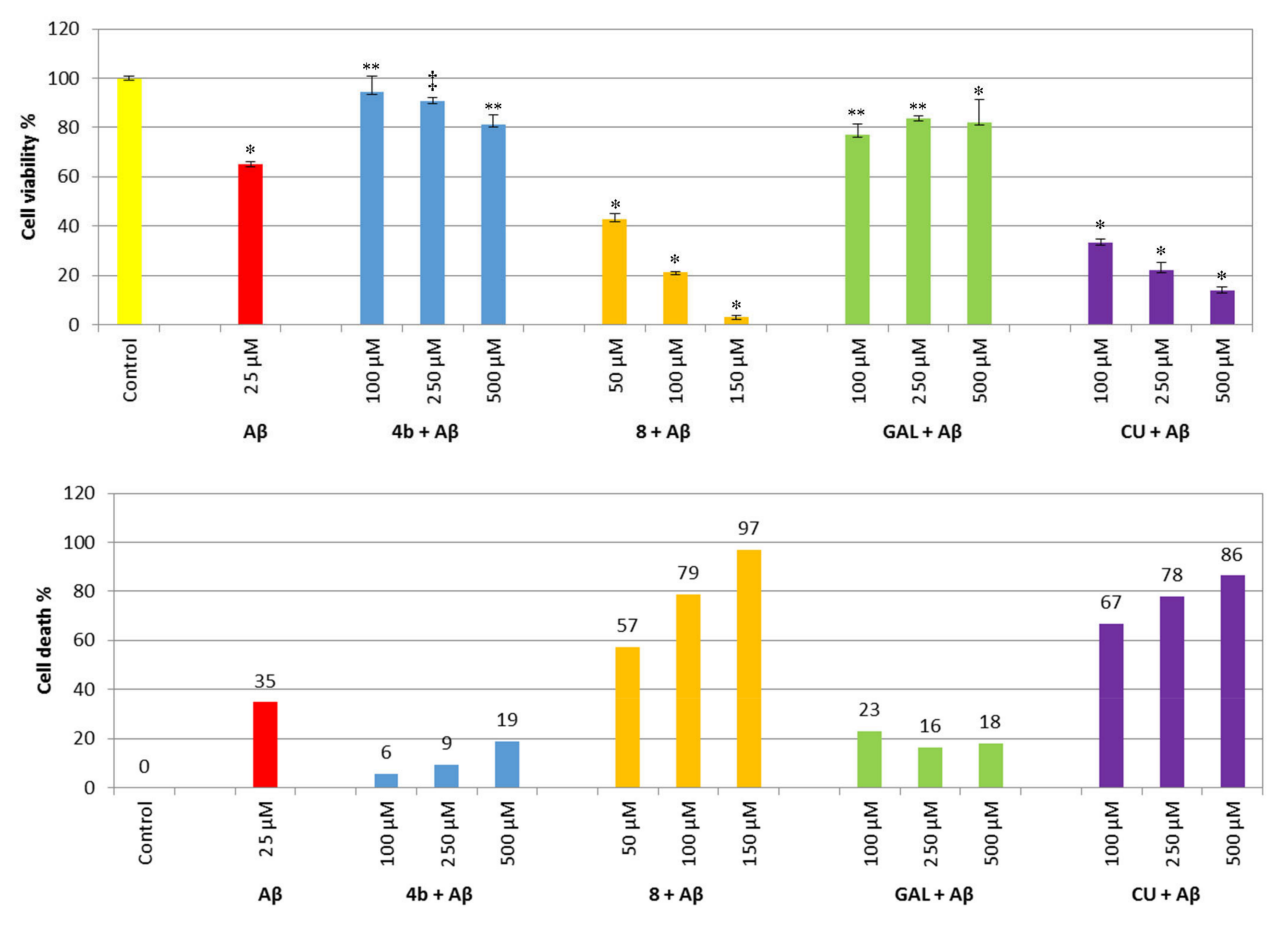
Cell viability (percentage) (up) and complementary cell death (percentage) (down) of SH-SY5Y cells treated for 48 h at 37 °C with 25 µM Aβ peptides and different concentrations of **4b**, **8**, GAL, and CU. Values are expressed as a mean ± SE, *n* = 3, *p*-values are presented as: * for *p* < 0.01, ** for *p* < 0.05, and ‡ for *p* ≤ 0.1.

According to the first scenario, designed in the present study, Aβ peptides were given at a concentration of 25 µM simultaneously with the tested AChE inhibitors at different concentrations and incubated for 48 h at 37 °C. The results for the cell viability and the complementary cell death after 48 h are given in Figure 4.

Compound **4b** provided the highest cell protection at 100 µM, where the cell viability decreased only by 6%. For compound **8**, the highest protection was achieved at 50 µM, but only 43% of the cells survived. GAL protected 84% of the cells at a concentration of 250 µM. CU achieved the highest cell viability of 33% at a concentration of 100 µM.

Immediate cell protection from Aβ toxicity was observed by **4b** and GAL. Compound **8** and CU potentiated the Aβ toxicity at the tested concentrations. The protection by **4b** and GAL can be observed morphologically in Figure 3. The cells treated with **4b** and GAL displayed better confluence than those treated by compound **8** and CU, where shape rounding and detachment from the surface was observed.

### 2.4. Cytotoxicity of Amyloid β Peptides Given after the Tested AChE Inhibitors

**Figure 5:**
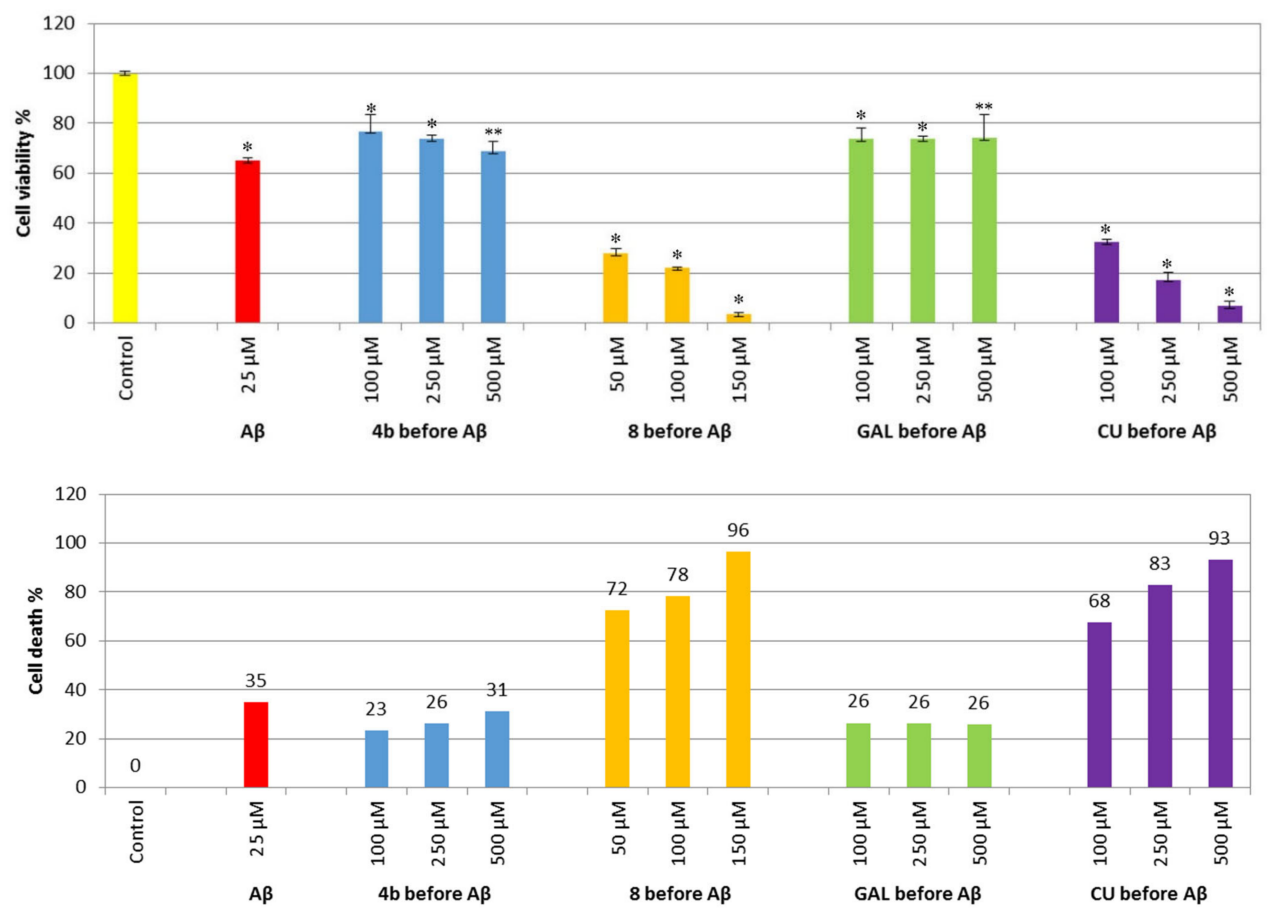
Cell viability (percentage) (up) and complementary cell death (percentage) (down) of SH-SY5Y cells treated for 24 h at 37 °C with different concentrations of **4b**, **8**, GAL, and CU, followed by incubation for 48 h at 37 °C with 25 µM Aβ peptides. Values are expressed as a mean ± SE, *n* = 3, *p*-values are presented as: * for *p* < 0.01, and ** for *p* < 0.05.

In order to assess the ability of the tested AChE inhibitors to prevent or diminish Aβ cytotoxicity, the cells were pre-incubated with the tested compounds for 24 h, followed by incubation with Aβ at 37 °C for 48 h. The results in this scenario are given in Figure 5.

Compound **4b** and GAL decreased the cytotoxicity caused by Aβ peptides applied after the tested compounds. For **4b**, the protection was the highest at 100 µM and reached 77% cell viability, 17% less than that of **4b** given simultaneously with Aβ. For GAL, the three tested concentrations provided the same protection of 74% viable cells, 3% to 10% less compared with simultaneous treatment. Here, again, compound **8** and CU failed to protect the cells from the toxic effect of Aβ peptides. In the second scenario, cells treated with **4b** and GAL demonstrated better confluence than those treated with compound **8** and CU, which corresponds well to the cytotoxicity test (Figure 3).

### 2.5. Cytotoxicity of Amyloid β Peptides Given before the Tested AChE Inhibitors

**Figure 6:**
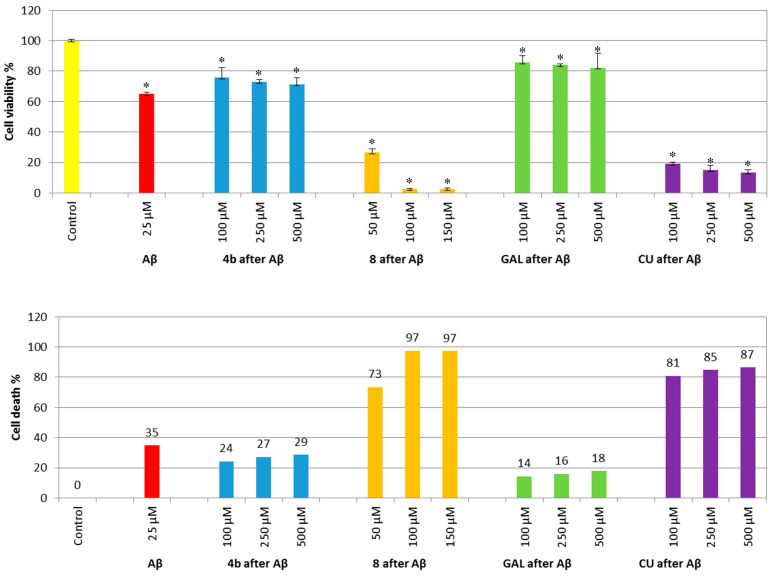
Cell viability (percentage) (up) and complementary cell death (percentage) (down) of SH-SY5Y cells treated for 24 h at 37 °C with 25 µM Aβ peptides followed by incubation for 48 h at 37 °C with different concentrations of **4b**, **8**, GAL, and CU. Values are expressed as a mean ± SE, *n* = 3, *p*-values are presented as: * for *p* < 0.01.

The third scenario was scheduled to test the ability of the AChE inhibitors to restore cell viability after the damage caused by Aβ. For this purpose, the cells were pre-incubated with Aβ for 24 h at 37 °C and then treated with the tested inhibitors for 48 h. The cell revival is illustrated in Figure 6.

Here, again, GAL and **4b** performed better than compound **8** and CU. Compound **4b** at a concentration of 100 µM restored 76% of the cells, while GAL reached 86% viability at the same concentration. More pronounced changes in cell morphology (rounding) and separation of the monolayer from the surface were observed for compound **8** and CU (Figure 3).

## 3. Discussion

Aβ_1–40_ and Aβ_1–42_ peptides are water soluble components of human plasma and cerebrospinal fluid, and under normal circumstances undergo regular clearance by phagocytosis in microglia [15]. For unknown reasons, these peptides form an unusual β-turn conformation in the central residues and convert into insoluble misfolded monomers that aggregate into neurotoxic oligomers [4]. One of the possible mechanisms of amyloid cytotoxicity is the formation of transmembrane channels leading to excessive Ca^2+^ influx, mitochondrial damage, and cell apoptosis [22]. Free radical damage caused by Aβ through increased H_2_O_2_ accumulation in cells is another possible mechanism of Aβ cytotoxicity [23].

It was found that GAL protects against Aβ cytotoxicity at concentrations of 25 µM to 1000 µM [10]. In the present study, this protective effect was confirmed—in the range 100–500 µM, GAL reduced the cell death caused by Aβ from 35% to 16–23% when administered simultaneously with the peptide (Figure 4). When the cells were pre-incubated with GAL, the effect was similar (Figure 5). More profound was the effect of GAL when it was administered after a pre-incubation with Aβ—the cells revived from 65% to 86% (Figure 6).

The cytoprotective effect of GAL is due to its ability to allosterically stimulate α7 nAChRs [9,24]. Alpha-7 nAChRs are associated with a higher Ca^2+^ permeability compared with other nAChRs [25]. The stimulation of nAChRs enhances and improves cognition [10]. In the presence of α-bungarotoxin, an α7-specific nAChR antagonist, the protective effect of GAL disappears [9].

CU inhibits the aggregation of Aβ peptides [26,27,28] by intercalation between them and β-sheet breaking [29,30,31,32,33,34,35]. In the present study, it was observed that CU does not protect against Aβ toxicity. Even more, CU potentiated the toxic effects of Aβ in all three scenarios—treatment simultaneously with Aβ, before introduction of Aβ, and after introduction of Aβ (Figure 4, Figure 5 and Figure 6). Obviously, the anti-aggregation activity of CU is not important for the cell survival. Similarly, the free radical damage caused by Aβ peptides also could be rejected as a putative reason for SH-SY5Y cell death as the antioxidant properties of CU do not help for cell protection.

**Figure 7:**
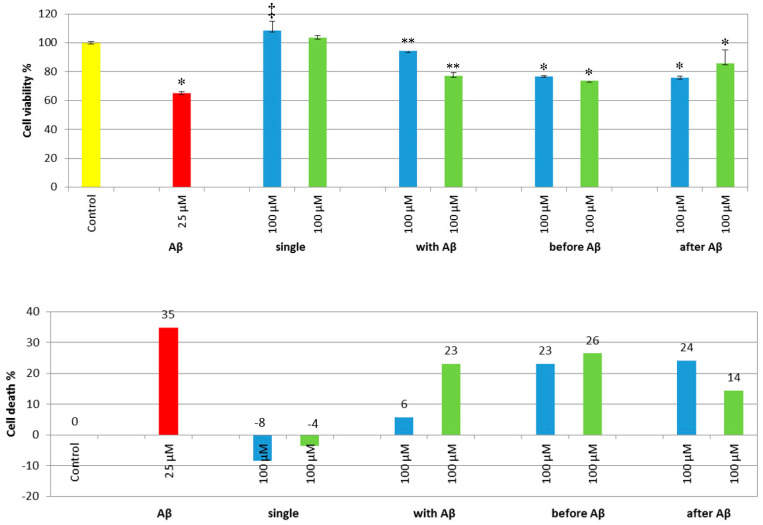
Cell viability (percentage) (up) and complementary cell death (percentage) (down) of SH-SY5Y cells treated with **4b** (blue bars) and GAL (green bars) alone, and by **4b** and GAL administered simultaneously with Aβ, before the introduction of Aβ, and after the introduction of Aβ. Values are expressed as a mean ± SE, *n* = 3, *p*-values are presented as: * for *p* < 0.01, ** for *p* < 0.05, and ‡ for *p* ≤ 0.1.

The newly designed and tested AChE inhibitors **4b** and **8** are both GAL derivatives. The compound **4b** is a hybrid of GAL and CU. One could expect that these GAL derivatives would have cytoprotective effects similar to those of GAL. The experiments in the present study showed that only **4b** behaved as GAL. Compound **8**, like CU, potentiated the cytotoxic effects of Aβ in all three scenarios. The comparison between **4b** and GAL revealed that **4b** protects better than GAL when administered simultaneously with Aβ (Figure 7). In the before-Aβ scenario, **4b** was slightly better than GAL, while in the after-Aβ test, GAL restored the damaged cells to a higher extent. The similar effects suggest a similar mode of action—**4b** might act as an allosteric modulator of α7 nAChRs. Recently, we found that **4b** is a low-toxic compound (LD_50_ in mice was 49 mg/kg), inhibits AChE in vivo and in vitro better than GAL, and demonstrates high antioxidant activity in vitro and ex vivo outperforming the activities of both GAL and CU [21]. Together with the findings in the present study, **4b** has emerged as a promising multi-target agent for a complex treatment of neurodegenerative disorders.

## 4. Materials and Methods

### 4.1. Materials

In the present study, the following materials were used: galantamine HBr (Galen-N Ltd., Sofia, Bulgaria, Mw = 368.3 g/mol, purity > 98%), curcumin (BioXtract, Belgium, Mw = 368.4 g/mol, purity > 98%), bovine serum albumin (Sigma-Aldrich, Taufkirchen, Germany, Mw ≈ 66 kD, purity > 96%), thiobarbituric acid (Sigma-Aldrich, Germany, Mw = 144.15 g/mol, purity > 98%), trichloroacetic acid (Sigma-Aldrich, Germany, Mw = 163.39 g/mol, purity > 99%), acetylthiocholine iodide (Sigma-Aldrich, Germany, Mw = 289.18, purity > 98%), 2,2-dinitro-5,5 dithiodibenzoic acid (DTNB) (Sigma-Aldrich, Germany, Mw = 396.35, purity > 98%), ethylenediaminetetraacetic acid (EDTA) (Sigma-Aldrich, Germany, Mw = 292.24, purity > 99%). 2,2′-Diphenyl-1-picrylhydrazyl (DPPH), 2,2′-azinobis-(3-ethylbenzothia- zine-6-sulfonic acid) (ABTS), 6-hydroxy-2,5,7,8-tetramethylchroman-2-carboxylic acid (Trolox), 2,4,6-tripyridyl-s-triazine (TPTZ), FeCl_3_·6H_2_O, sodium acetate, potassium persulphate, and butylhydroxy toluol (BHT) were purchased from Sigma-Aldrich. 1,1,1,3,3,3-Hexafluoro-2-propanol (HFP) was purchased from Sigma-Aldrich. Amyloid beta peptide (1–42) (human, Aβ_1–42_) was purchased from Abcam (UK). All other chemicals, including the solvents, were of analytical grade.

The synthesis of compounds **4b** and **8** along with detailed analytical data has already been reported in the context of the synthesis of a series of new GAL derivatives [16,17].

**Scheme 1:**
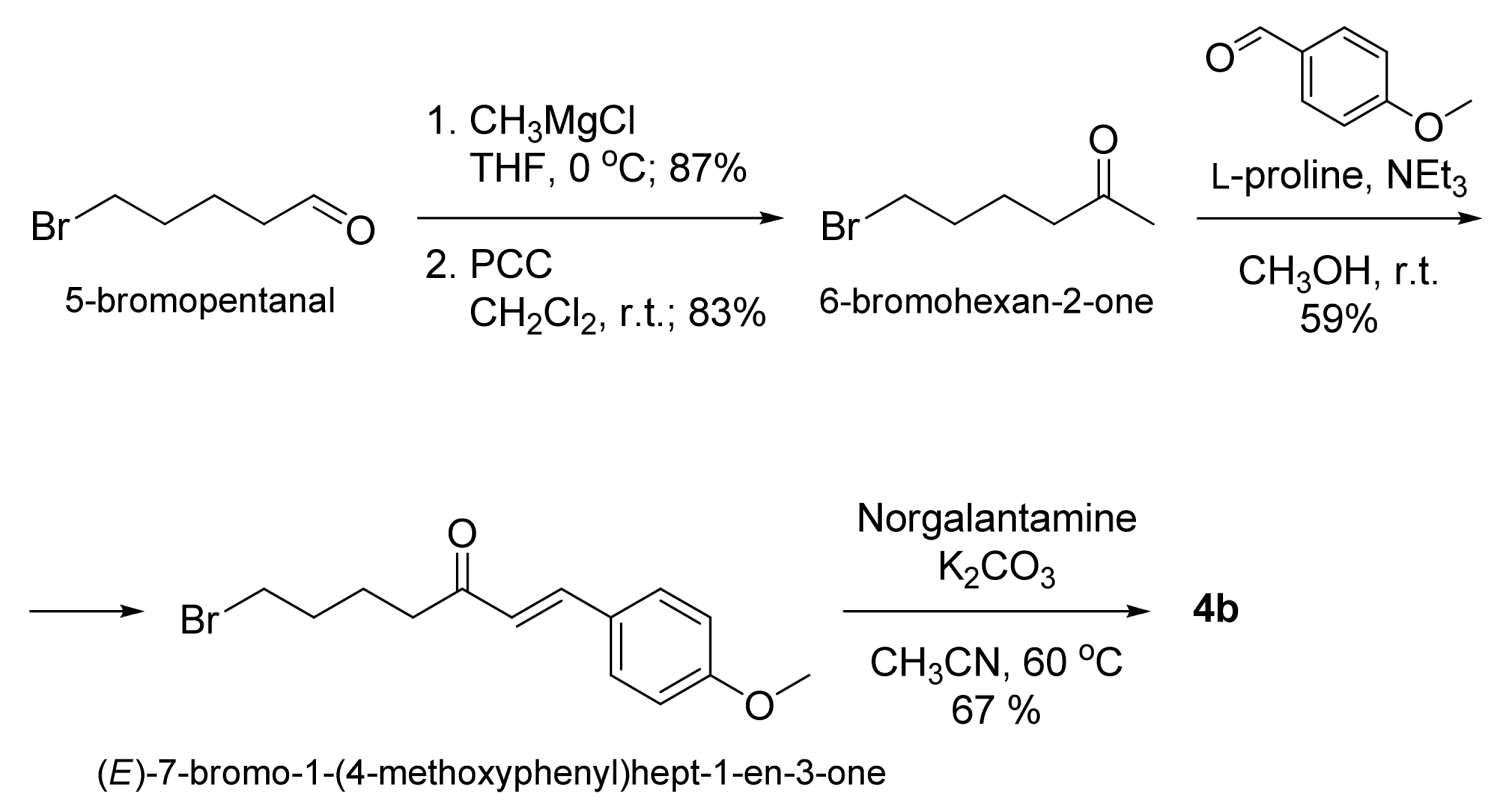
Synthesis of compound **4b**.

The synthesis of **4b** involved the construction of a bromo-containing linker and its substitution with norgalantamine (Scheme 1). Thus, monobromination and mild oxidation of 1,5-pentandiol afforded 5-bromopentanal, which was subsequently attacked by Grignard reagent and oxidized to 6-bromohexan-2-one. This ketone was subjected to mild Aldol condensation with *p*-methoxybenzaldehyde to give the desired bromo-linker. The nucleophilic substitution with norgalantamine proceeded in the presence of K_2_CO_3_ as a base to give compound **4b** in moderate yield (Scheme 1). The analytical data of compound **4b** are given in Supplementary Materials.

**Scheme 2:**
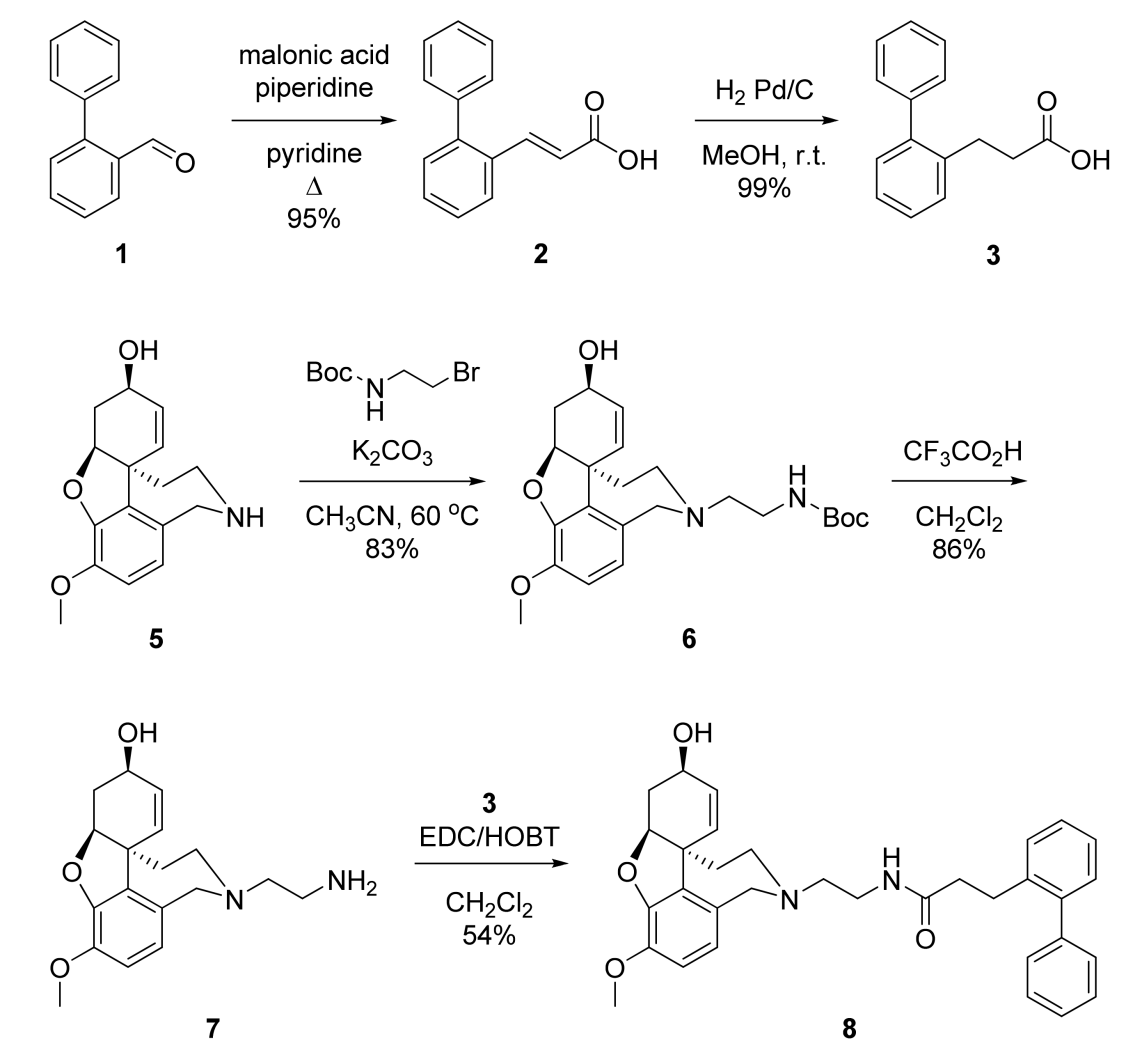
Synthesis of compound **8**.

The synthesis of compound **8** involved the construction of two building blocks, 3-(biphenyl-2-yl)propanoic acid **3** and an amino-derivative of GAL **7**, and their subsequent amide coupling (Scheme 2). The desired acid **3** was prepared via Knoevenagel condensation of biphenyl-2-carbaldehyde with malonic acid to (*E*)-3-(biphenyl-2-yl)acrylic acid **2**, which was quantitatively reduced. The second building block **7** was synthesized via nucleophilic substitution of *tert*-butyl *N*-(2-bromoethyl)carbamate with norgalantamine **5** to give GAL-derivative **6**, which was subjected to Boc-deprotection. The amide coupling was performed with the reagents for peptide synthesis *N*-ethyl-*N′*-(3-dimethylaminopropyl)-carbodiimide hydrochloride (EDCI) and 1-hydroxy-benzotriazole hydrate (HOBT) to give compound **8** in moderate yield. The analytical data of compound **8** are given in Supplementary Materials.

### 4.2. Preparation of Aβ Peptide

Amyloid-β peptide (Aβ_1–42_) was dissolved in HFIP incubated for 1h at room temperature and aliquoted for storage at −80 °C until usage. For cytotoxicity experiments, Aβ_1–42_ aliquots were evaporated and dissolved in DMSO and a PBS buffer, pH 7.4 [36,37].

### 4.3. Cytotoxicity Test

SH-SY5Y cells (human neuroblastoma cell line, ATCC CRL-2266) were seeded onto a 96-well plate at a density of 3 × 10^4^ cells per well in DMEM F12 medium, supplemented with 15% FCS (fetal calf serum), 100 U/mL penicillin, 100 mg/mL streptomycin, and 1% amino acids. After 24 h preincubation at 37 °C in a humidified atmosphere of 5% CO_2_, the culture media was replaced with fresh DMEM medium without FCS, supplemented with different concentrations of: compound **4b** (25, 50, 100, 250, and 500 μM); compound **8** (25, 50, 100, 250, and 500 μM); curcumin (25, 50, 100, 250, 500, and 1000 μM); galantamine hydrobromide (25, 50, 100, 250, 500, and 1000 μM); and Aβ_1–42_ in concentrations of 5, 10, 25, and 50 μM, respectively. After 48 h at 37 °C, SH-SY5Y cells, treated with different concentrations of the individual compounds, were washed with PBS and incubated with MTT (3-(4,5-dimethylthiazol-2-yl)-2,5-diphenyltetrazolium bromide) reagent (0.5 g/mL) in serum-free DMEM medium for 3 h at 37 °C. The MTT formazan product was dissolved in DMSO and the absorbance was detected immediately with an Epoch™ Microplate Spectrophotometer (BioTek) at 570 nm [38]. Cell viability was presented as the ratio: (absorbance of the treated wells)/(absorbance of the control wells) × 100%.

Each experiment was performed three times. Values were expressed as a mean ± SE. The *p*-value was calculated using the Student’s *t*-test. Data with *p* < 0.01 were considered as highly significant, with *p* < 0.05 considered as statistically significant and with *p* ≤ 0.1 as marginally significant.

In order to study the combined effects of Aβ peptides (used in all combined treatments at a constant concentration of 25 μM) and various concentrations of tested compounds (GAL, CU, **4b**, or **8**) on the viability of SH-SY5Y cells (3 × 10^4^/well), we applied the following experimental scenarios:

− Simultaneous administration of Aβ (25 μM) and each of the tested compounds in different concentrations and incubation at 37 °C for 48 h
− Preincubation of SH-SY5Y cells with each of the tested compounds in different concentrations for 24 h, followed by the addition of Aβ (25 μM) and incubation at 37 °C for 48 h (the before Aβ scenario)
− Initial preincubation of SH-SY5Y cells with Aβ (25 μM) for 24 h, then with each of the tested compounds in different concentrations and incubation at 37 °C for 48 h (the after Aβ scenario).

Morphological changes were visualized at regular time intervals (24 h) via microscopy observations (Nikon Eclipse microscope—objective lens 25×).

## Supplementary Materials

The following are available online at https://www.mdpi.com/article/10.3390/ijms22147592/s1. 1. Analytical data of compound **4b**. 2. Analytical data of compound **8**.
