# Supplementary material for: A Galantamine–Curcumin Hybrid Decreases the Cytotoxicity of Amyloid-Beta Peptide on SH-SY5Y Cells
Source: Int J Mol Sci. 2021 Jul 15;22(14):7592. doi: 10.3390/ijms22147592 (PMC8307467; doi:10.3390/ijms22147592)
Supplement: Supplementary file 1 [file ijms-22-07592-s001.zip › ijms-1277884-supplementary.pdf]

Supplementary Material

Contents:

1. Analytical data of compound **4b**.
2. Analytical data of compound **8**.

1. Analytical data of compound **4b**.

Yield: 51%; white crystals; m.p. 44-46°C. $[\alpha]^{20}_{D}$ = -84.4 (c 1.000, $CHCl_3$). ^{1}H NMR ($CDCl_3$, 600 MHz) δ = 7.50 (d, *J* = 16.1 Hz, 1H, H-1′), 7.49 (d, *J* = 8.7 Hz, 2H, arom.), 6.91 (d, *J* = 8.8 Hz, 2H, arom.), 6.65 (d, *J* = 8.2 Hz, 1H, H-2), 6.62 (d, *J* = 8.2 Hz, 1H, H-1), 6.61 (d, *J* = 16.1 Hz, 1H, H-2′), 6.08 (d, *J* = 10.2 Hz, 1H, H-8), 6.00 (dd, *J* = 10.2, 4.6 Hz, 1H, H-7), 4.61 (br, 1H, H-6), 4.14-4.12 (m, 1H, H-4a), 4.13 (d, *J* = 15.3 Hz, 1H, H-12), 3.85 (s, 3H, OCH_3), 3.84-3.81 (m, 1H, H-12), 3.82 (s, 3H, OCH_3), 3.35 (t, *J* = 13.7 Hz, 1H, H-10), 3.18 (d, *J* = 14.9 Hz, 1H, H-10), 2.70-2.66 (m, 1H, H-5), 2.65 (t, *J* = 7.3 Hz, 1H, H-4′), 2.58-2.52 (m, 1H, H-7′), 2.55-2.48 (m, 1H, H-7′), 2.43 (br, 1H, OH), 2.07-2.02 (m, 1H, H-9), 2.02-1.98 (m, 1H, H-5), 1.72-1.63 (m, 2H, H-5′), 1.57-1.52 (m, 3H, H-6′, H-9) ppm. ^{13}C NMR ($CDCl_3$, 150.9 MHz) δ = 200.24 (CO), 161.50 (C arom.), 145.74 (C-3a), 144.06 (C-3), 142.20 (C-1′), 133.08 (C-12b), 129.92 (2CH arom., C-12a), 127.57 (C-7), 127.10 (C arom.), 126.86 (C-8), 123.91 (C-2′), 122.04 (C-1), 114.36 (2CH arom.), 111.06 (C-2), 88.69 (C-6), 62.04 (C-4a), 57.64 (C-12), 55.83 (OCH_3), 55.37 (OCH_3), 51.53 (C-10), 50.97 (C-7′), 48.35 (C-8a), 40.49 (C-4′), 32.87 (C-9), 29.89 (C-5), 26.87 (C-6′), 22.14 (C-5′) ppm. HRMS found for $C_{30}H_{36}NO_5$: m/z 490.2587 $[M+H]^+$, calcd. m/z 490.2588.

2. Analytical data of compound **8**.

Yield: 54%. [α]D 20 = -56.7 (c 0.4115, CHCl3). 1H-NMR (CDCl3, 600 MHz): δ = 7.42–7.40 (m, 2H, arom.), 7.36–7.34 (m, 1H, arom.), 7.32–7.28 (m, 3H, arom.), 7.27–7.24 (m, 2H, arom.), 7.22–7.21 (m, 1H, arom.), 6.66 (d, J = 8.2 Hz, 1H, H-2), 6.58 (d, J = 8.2 Hz, 1H, H-1), 6.06 (dd, J = 10.4, 0.8 Hz, 1H, H-8), 6.02 (dd, J = 10.2, 4.7 Hz, 1H, H-7), 5.78 (br, 1H, NH), 4.60 (br, 1H, H-6), 4.15 (br, 1H, H-4a), 4.09 (d, J = 15.6 Hz, 1H, H-12), 3.83 (s, 3H, OCH3), 3.65–3.63 (m, 1H, H-12), 3.63 (t, J = 13.8 Hz, 1H, H-10), 3.24–3.14 (m, 2H, CH2NHCO), 3.03 (d, J = 14.7 Hz, 1H, H-10), 2.97 (t, J = 7.8 Hz, 2H, COCH2), 2.71–2.68 (m, 1H, H-5), 2.56–2.55 (m, 1H, NCH2), 2.50–2.46 (m, 1H, NCH2), 2.25 (t, J = 8.0 Hz, 2H, COCH2CH2), 2.02–1.94 (m, 2H, H-5, H-9), 1.51 (d, J = 12.6 Hz, 1H, H-9) ppm. 13C-NMR (CDCl3, 150.9 MHz) δ = 172.21 (CO), 145.83 (C-3a), 144.28 (C-3), 141.79 (C, arom.), 141.52 (C, arom.), 138.09 (C, arom.), 132.93 (C-12b), 130.18 (CH, arom.), 129.26 (CH, arom., C-12a), 129.02 (2CH, arom.), 128.23 (2CH, arom.), 127.83 (C-7), 127.53 (CH, arom.), 126.95 (CH, arom.), 126.50 (C-8), 126.20 (CH, arom.), 122.11 (C-1), 111.12 (C-2), 88.64 (C-6), 61.96 (C-4a), 56.81 (C-12), 55.85

(OCH3), 51.54 (C-10), 48.92 (NCH2), 48.34 (C-8a), 37.48 (COCH2CH2), 35.95 (CH2NHCO), 32.79 (C-9), 29.86 (C-5), 29.22 (COCH2) ppm. C33H36N2O4 (524.65): calcd. C 75.55, H 6.92, N 5.34, found C 75.23, H 7.09, N 5.06.
